# On the importance of cavity-length and heat dissipation in GaN-based vertical-cavity surface-emitting lasers

**DOI:** 10.1038/srep09600

**Published:** 2015-04-15

**Authors:** W. J. Liu, X. L. Hu, L. Y. Ying, S. Q. Chen, J. Y. Zhang, H. Akiyama, Z. P. Cai, B. P. Zhang

**Affiliations:** 1 Department of Physics, Xiamen University, Xiamen 361005, P. R. China; 2 Department of Electronic Engineering, Xiamen University, Xiamen 361005, P. R. China; 3 Department of Electronic Engineering, East China Normal University, 500 Dongchuan Road, Shanghai 200241, China; 4 Institute for Solid State Physics, University of Tokyo, 5-1-5 Kashiwanoha, Kashiwa, Chiba 277-8581, Japan

## Abstract

Cavity-length dependence of the property of optically pumped GaN-based vertical-cavity surface-emitting lasers (VCSELs) with two dielectric distributed Bragg reflectors was investigated. The cavity lengths were well controlled by employing etching with inductively coupled plasma and chemical mechanical polishing. It was found that the lasing characteristics including threshold, slope efficiency and spontaneous emission coupling factor were substantially improved with reducing the cavity length. In comparison with the device pumped by a 400 nm pulsed laser, the lasing spectrum was featured by a red shift and simultaneous broadening with increasing the pumping energy of a 355 nm pulsed laser. Moreover, the lasing threshold was much higher when pumped by a 355 nm pulsed laser. These were explained by taking into account of the significant heating effect under 355 nm pumping. Our results demonstrate that a short cavity length and good heat-dissipation are essential to GaN-based VCSELs.

Over the past few years, GaN-based vertical-cavity surface-emitting lasers (VCSELs) have been attracting much attention owing to their superior properties over edge-emitting lasers and potential applications in high-density optical storage, large area laser displays, lighting devices, etc. Both optically pumped and electrically pumped GaN-based VCSELs have been realized^1^,^2^,^3^,^4^,^5^,^6^,^7^,^8^,^9^,^10^,^11^,^12^,^13^,^14^. The VCSEL structure can be mainly concluded into two types. One is the hybrid distributed Bragg reflector (DBR) VCSEL structure combining an epitaxially grown DBR and a dielectric DBR^1^,^2^,^3^,^4^. In this case, the most challenging problem is the preparation of high reflectivity nitride-based DBR with smooth epitaxial surface due to the large lattice mismatch between GaN and AlN (AlGaN). Besides, the crystalline quality of the quantum wells grown on such DBRs usually deteriorates. Another structure is dielectric DBR VCSEL that contains two dielectric DBRs^6^,^7^,^8^,^9^. For this structure, the preparation of high reflectivity dielectric DBR is much easier in comparison with the epitaxially grown DBRs. In fabrication of dielectric DBR VCSEL, bonding and laser lift-off techniques are usually employed.

For GaN-based microcavity devices, the change of cavity length can strongly affect the spontaneous emission, the cavity gain, the quality factor *etc*. In VCSEL, this should influence the lasing characteristics. Investigation of VCSELs with different cavity lengths is important to further explore physical mechanism inside the cavities and has a guiding significance on the realization of high performance GaN-based VCSELs. So far, however, no experimental study of the properties among VCSELs with different cavity lengths has ever been reported. In this letter, the lasing properties in dielectric DBR VCSELs with cavity lengths ranging from 8.5 *λ* to 18 *λ* (*λ* is the peak wavelength) is studied. Under the pumping by a 400 nm pulsed laser, the lasing spectrum was stable against the variation of pumping energy. With reducing the cavity length, the lasing threshold decreased dramatically and meanwhile, the spontaneous emission coupling factor, slope efficiency and lasing linewidth increased. However, red shift of the lasing wavelength and linewidth broadening were observed when pumped by 355 nm laser as the pumping energy was increased. In addition, the lasing threshold became higher compared with the case of 400 nm pumping. These were explained by heating effect.

## Results

**Figure 1:**
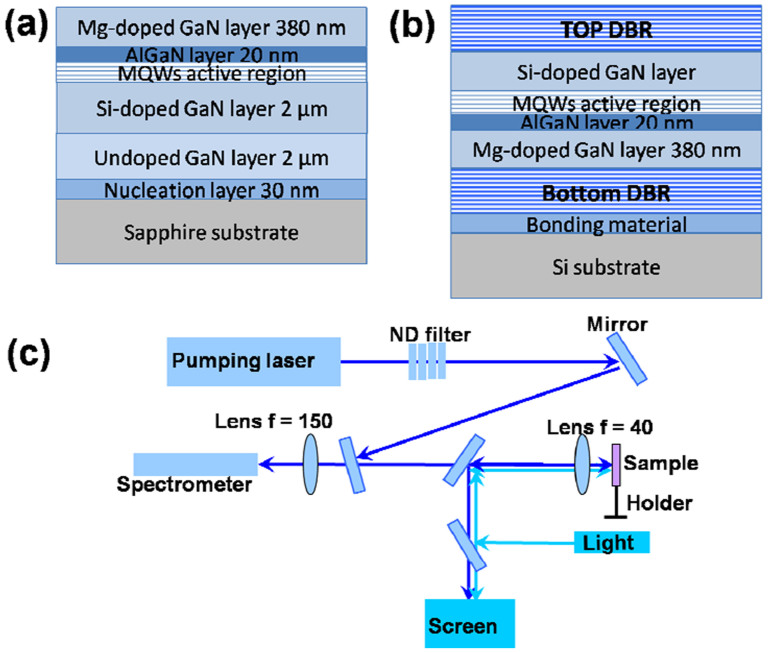
Schematic structure of the (a) epitaxial layer and (b) GaN-based VCSEL with dielectric DBRs. (c) Diagrams of the experimental setup for optical pumping measurements.

The schematic diagram of the epitaxial layer and the GaN-based VCSEL are shown in figures 1(a) and (b), respectively. The device fabrication process was similar to our previous study^15^. Different cavity lengths of the VCSELs were obtained through varying the thickness of the n-GaN layer. Specifically, after removal of sapphire substrate, etching with inductively coupled plasma (ICP) was carried out for different times to obtain three samples with different n-GaN thicknesses. Followed by chemical mechanical polishing (CMP) and deposition of the top DBR, three samples with cavity lengths of 18 *λ*, 11.5 *λ* and 8.5 *λ* (including penetration depth of both DBRs) were obtained, which are, for convenience here, defined as sample S_1_, S_2_, and S_3_, respectively. The root mean square (RMS) roughness of the GaN surface measured by atomic force microscopy (AFM) was 0.3 nm in a scan area of 2 μm × 2 μm after CMP. The sub-nanometer roughness smooth surface provided favorable condition for high reflectivity DBRs evaporation^15^. The experimental setup for optical measurements is shown in figure 1(c). The sample was vertically placed and clamped on a holder without cooling treatment. All the experiments were performed at room temperature.

**Figure 2:**
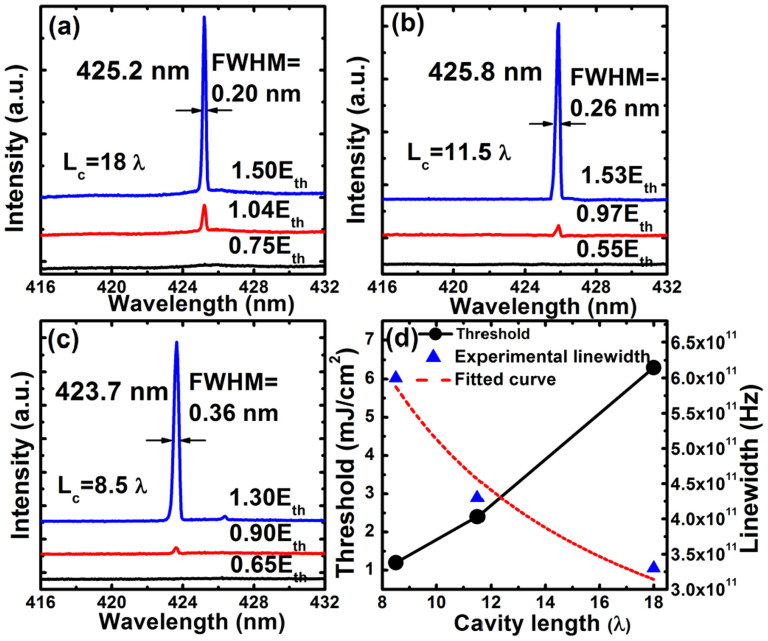
(a), (b) and (c) are the emission spectra at various pumping energies for samples S_1_, S_2_, S_3_ respectively. (d) Lasing threshold and lasing linewidth as a function of optical cavity length.

Figure 2(a), (b) and (c) show the emission spectra of S_1_, S_2_, and S_3_ at different pumping energies of a 400 nm pulse laser at room temperature. It is known that when cavity length is changed, the position of resonant mode moves. Because of the large cavity length, for each sample there are a few resonant modes within the high-reflectance band of the DBR. For comparison, peaks having similar wavelengths, around 425 nm, were chosen in this study. It can be seen in each spectrum that sharp peak emerged at around 425 nm and grew dramatically above the threshold. For S_1_, lasing action was achieved with a threshold pumping energy density of 6.3 mJ/cm^2^ (corresponds to the lasing energy of 120 nJ/pusle), while the threshold pumping energy density of S_2_ was 2.2 mJ/cm^2^ (corresponds to the lasing energy of 42 nJ/pusle). For the shortest-cavity-length sample S_3_, the threshold pumping energy density decreased remarkably to 1.2 mJ/cm^2^ (corresponds to the lasing energy of 23 nJ/pusle). The S_1_, S_2_ and S_3_ had the lasing linewidths of 0.20 nm, 0.26 nm and 0.36 nm, corresponding to the frequency linewidths of 3.3 × 10^11^ Hz, 4.3 × 10^11^ Hz and 6.0 × 10^11^ Hz, respectively. It was then concluded in figure 2(d) that, with reducing the cavity length, the lasing threshold decreased dramatically, and meanwhile, the lasing linewidth increased.

**Figure 3:**
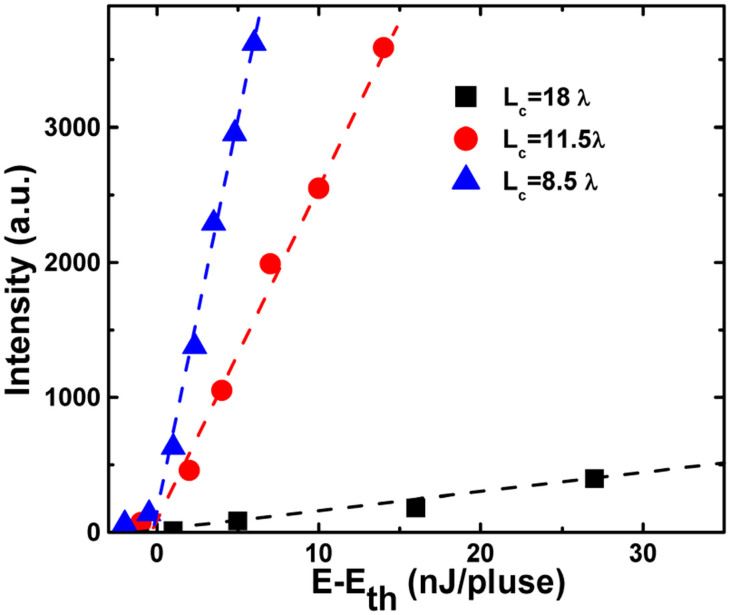
Lasing intensity above the threshold as a function of pumping energy.

Figure 3 shows the comparison of lasing intensity above threshold as a function of pumping energy of the three samples. Here relative slope efficiency can be evaluated by the slope of the line in figure 3. It was seen that the efficiency increased rapidly with reducing the cavity length. Supposing the value of the relative slope efficiency in S_1_ equals to 1, then the values of that in S_2_ and S_3_ were 11 and 15, respectively. Therefore, the slope efficiency in short-cavity-length VCSEL is much larger than that in long-cavity-length VCSEL. One reason for this is attributed to the reduced internal loss inside the cavity with reducing the cavity length. The other reason is due to the enhanced cavity gain coefficient which will be discussed later.

**Figure 4:**
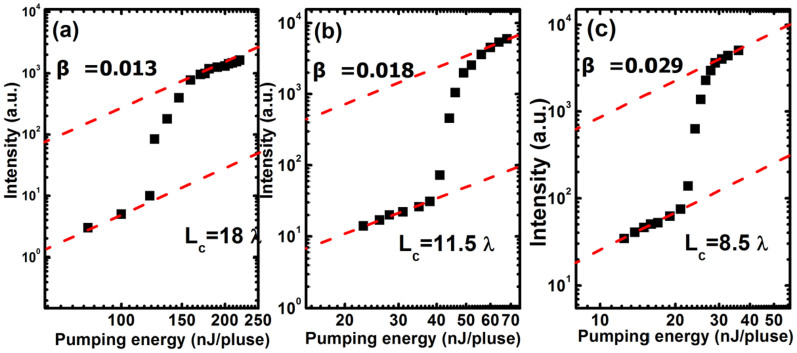
(a), (b) and (c) are emission intensities as a function of pumping energy plotted in double logarithmic scale. The lines are guides for the eye.

The spontaneous emission factor *β*, which reflects the coupling efficiency of the spontaneous emission to the lasing mode, plays an important role to evaluate the performance of the VCSEL. In order to understand the spontaneous emission factor *β* in GaN-based VCSELs with different cavity length, we plotted the emission intensities of the three samples in a double logarithmic scale as shown in figure 4(a), (b) and (c). The *β* value is corresponding to the difference between the heights of the emission intensities before and after lasing^16^. The estimated *β* values in VCSELs with cavity length of 18 *λ*, 11.5 *λ* and 8.5 *λ* were 1.3 × 10^−2^, 1.8 × 10^−2^ and 2.9 × 10^−2^, respectively. *β* is in the order of 10^−5^ for edge-emitting semiconductor lasers. This means that *β* in these VCSELs is effectively coupled 1000 times higher to the lasing mode compared with that of typical edge-emitting semiconductor lasers.

**Figure 5:**
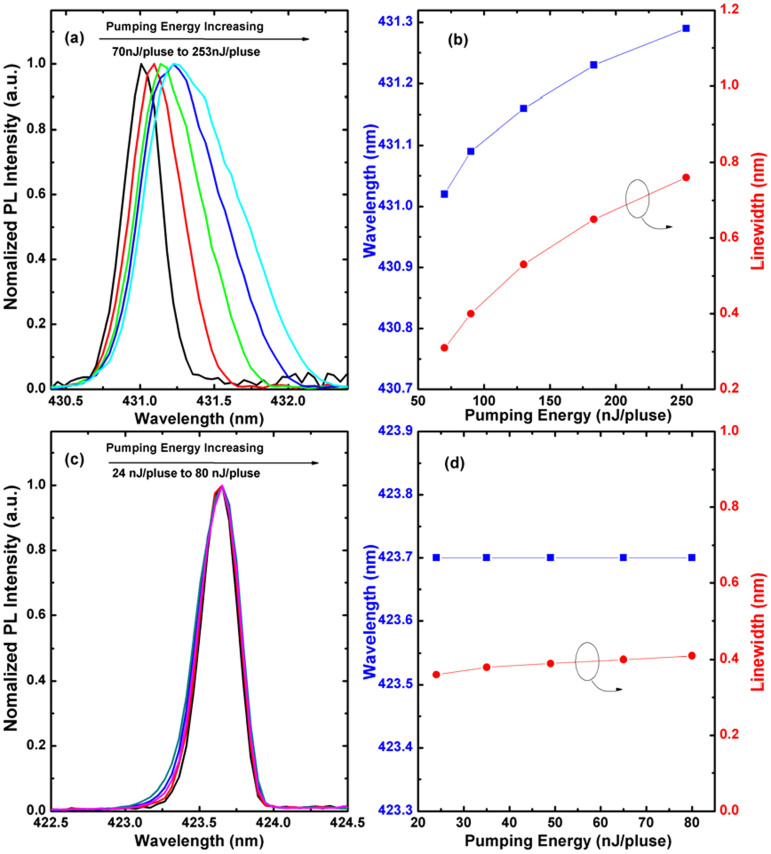
Normalized lasing spectra (a) (c) and lasing wavelengths and FWHM of the lasing spectra (b) (d), as a function of pumping energy pumped by the 355 nm laser and 400 nm laser, respectively.

Figure 5(a) shows the normalized lasing spectra of GaN VCSEL with cavity length of 8.5 *λ* pumped by the 355 nm laser. Red shift of the lasing wavelengths and linewidth broadening are clearly observed, as shown in figure 5(b). On the contrary, the lasing spectrum remains almost stable when pumped by the 400 nm laser, as shown in figure 5(c) and (d). In addition, the lasing threshold energy (E_th_ = 63 nJ/pulse, corresponds to an energy density of 3.2 mJ/cm^2^) when pumped by the 355 nm laser is a few times higher than that of the 400 nm laser (1.2 mJ/cm^2^). These results indicate that the pumping laser has a significant influence on the lasing properties of the VCSELs.

## Discussion

The increase of lasing frequency linewidth in figure 2(d) can be explained from the following equation^17^:
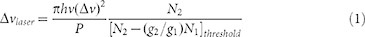


Where *ν* is the laser frequency, *P* is the output power, *N*_1_ and *N*_2_ are the population densities of the two energy levels involved in the induced transition, *g*_1_ and *g*_2_ are the degeneracy of the two energy levels. When pumped by the 400 nm laser, the lasing linewidth as well as the lasing wavelength remained almost stable with the pumping energy increased to three times of the threshold (Fig. 5(c) (d)). Therefore, the factor 
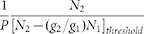
 is approximately as a constant value for the GaN-based VCSELs. As demonstrated in equation (1), the frequency width of the cavity resonance Δ*ν* strongly affects the lasing frequency linewidth. For a GaN-based Fabry-Perot cavity, the well-known imperfect crystalline quality of the GaN layer should generate extra internal losses in the cavity^15^,^18^. The Δ*ν* value changes with the cavity length, as given by the following equation^19^:
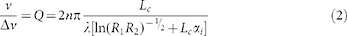
Where *Q* is the quality factor, *n* is the refractive index, *L_c_* is the cavity length, *α_i_* is the internal loss. The experimental results can be well described by the dashed curve in figure 2(d) according to equations (1) and (2). Then the internal loss *α_i_* caused by the GaN layer was estimated to be 149 cm^−1^ at the lasing wavelength of 425 nm. This value is similar to the reported result (about 160 cm^−1^ at 425 nm) in Ref. 20.

The variation of *β* values in Fig. 4 with respect to the cavity length can be analyzed based on the following equations^4^:
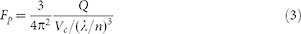
and
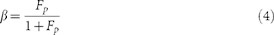


Where *F_p_* is the Purcell factor, and *V_c_* is the effective optical volume of the laser.

According to equations (2) and (3), *F_p_* can be rewritten as:
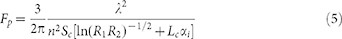


*S_c_* is defined as *V_c_*/*L_c_*, which represents the spot area of the laser emission. Thus, it was easily seen that the *F_p_* value increased with decreasing the cavity length. Consequently, the *β* value increased too.

The lasing threshold is a key parameter to evaluate the performance of semiconductor lasers. The decrease of lasing threshold with reducing cavity length is considered to be related to the following three aspects. First is the enhanced cavity gain coefficient with reducing the cavity length due to the cavity quantum electrodynamics effects. The gain characteristic is an important parameter of VCSELs since it has significant influence on lasing threshold. The enhanced cavity gain coefficient factor *κ* can be written as^21^:
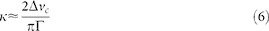


Where Γ is the linewidth of the spontaneous emission without a cavity, and Δ*ν_c_* is the frequency spacing between cavity modes which can be expressed as:
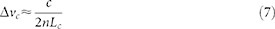


and so yields
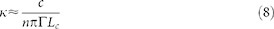


It is obvious that the *κ* factor increased inversely with reducing the cavity length, leading to the significantly enhanced cavity gain and thus reduced lasing threshold and increased slope efficiency. For samples S1 and S3, the cavity length is reduced approximately a half, and *κ* factor is then doubled.

The second aspect is the increased spontaneous emission factor *β* value with reducing the cavity length, as discussed above. The *β* value reflects the coupling efficiency of spontaneous emission to the lasing mode. The higher *β* value represents a higher coupling efficiency, which decreases the lasing threshold. Experimental results demonstrate that, for samples S1 and S3 where the cavity length is reduced approximately a half, the *β* value is doubled.

The third aspect is the reduced absorption loss by the GaN layer at the lasing wavelength and the less absorption of the injected laser. Single pass optical loss due to a single layer can be calculated from^22^:
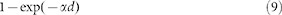


Where *α* is the absorption coefficient at the lasing wavelength (or at the injected laser wavelength), *d* is the thickness of the layer. The thickness of the removed n-type GaN layer was about 1.6 μm by comparing the thicknesses of S_1_ and S_3_. According to equation (9), this thickness corresponds to a single pass optical loss of 2.3% since the absorption coefficient *α_i_* is 149 cm^−1^ at the lasing wavelength of 425 nm. The absorption loss of the 400 nm injected laser intensity was considered to be a little higher than this value due to its shorter wavelength. A single pass reflection loss of 2.0% due to DBRs can be calculated from the expression 1 − exp(−*α_m_L_c_*), in which 
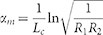
 and R_1_ = 99%, R_2_ = 97%. It is clear that the optical loss due to absorption by GaN is substantial.

From the discussions above, it is concluded that reducing the cavity length is a favorable way to decrease the lasing threshold of GaN-based VCSELs.

Now we discuss the behavior of the lasing performances of VCSELs under different pumping lasers. Compared with the case of 400 nm pumping, the lasing wavelength shifts to red and linewidth increases when pumped by the 355 nm laser with increasing the pumping energy, and the lasing threshold became higher. We consider these variations are caused by heating effect. First, under the pumping of the 355 nm laser where the photon energy of the laser is higher than the GaN bandgap, most of the photons were absorbed within the first hundreds nanometers by the GaN layer with an absorption coefficient of about 10^5^ cm^−1^ 20, resulting in a rapid increase of sample temperature. This heating effect of the 355 nm laser has been employed to the decomposition of GaN during laser lift-off process, where the sample temperature is expected to be higher than 850°C^23^,^24^. Second, a large number of carriers were generated in the GaN layer by the 355 nm laser, which would turn into heat during the transport process. In case of 400 nm laser pumping, however, the photon energy of the laser is smaller than the GaN bandgap and it can be absorbed in the InGaN well layers only and almost no transport process is necessary. Third, when pumped by a 355 nm laser, the quantum defect is much higher than that when pumped by a 400 nm laser. The high quantum defect also means a high thermal generation. As is known that, the DBRs formed by SiO_2_/Ta_2_O_5_, have extremely low thermal conductivity. Thus, considerable amounts of heat generated in GaN layer could not dissipated quickly. Instead, it accumulated to a high level and ultimately enlarged the cavity length, due to thermal expansion, and caused the red shift of the laser wavelength with increasing pumping energy. The elongate of the cavity length would increase the threshold value too. On the other hand, the increase of lasing FWHM can be explained by the aggravated interaction with phonons (or lattice vibration) as a result of heating. These results revealed that the performance of the VCSELs is strongly influenced by the heating effect. While in electrically injected GaN-based VCSELs, the heating effect could also be serious due to the high driving current density and large resistance, thus increasing the threshold current density. Therefore, excellent heat dissipation ability is crucial to realize the electrical pumped GaN-based VCSELs, which is consistent with previous reports^12^,^14^. Apart from the heating effect, pulse width of the pump laser may also play some role. At the same pumping energy, the laser with narrower pulse width could have a higher peak power. This means that a higher carrier density can be achieved instantaneously. In our experiments, pulse widths of the 355 nm and 400 nm pumping lasers were 25 ns and 150 fs, respectively, with a difference in the order of ~10^5^. It is clear that, for 400 nm pumping laser, a much higher carrier peak density is expected which may lead to a lower threshold.

**Figure 6:**
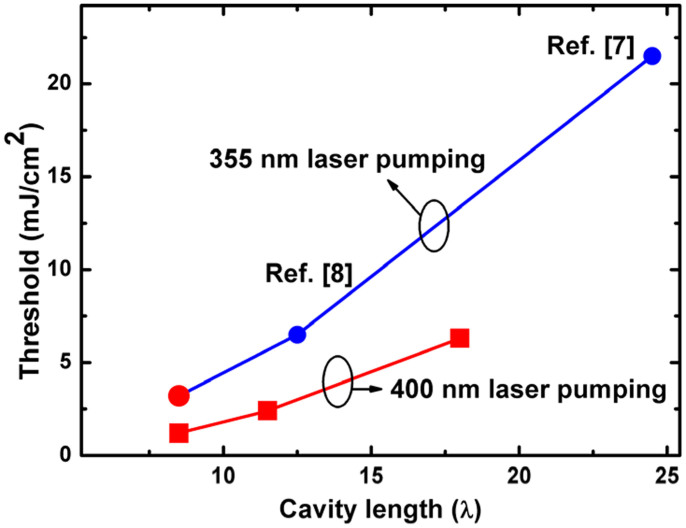
Lasing threshold of the GaN-based VCSELs as a function of the cavity length plotted together with other published results. The circle points were the results pumped by the 355 nm laser, and the solid squares were pumped by the 400 nm laser. Our results were marked red and the blue ones were taken from references.

Figure 6 shows the experimental results of the lasing threshold of the GaN-based VCSELs as a function of the cavity length obtained under different pumping lasers. The circle points were the results pumped by the 355 nm pulse laser including our data of sample S_3_ and the results taken from Ref. 7 and Ref. 8 that had similar VCSEL structures with ours. Also added in the figure (solid squares) were our data that pumped by the 400 nm pulse laser. It was seen that the lasing threshold measured by the 355 nm laser followed the same variation curve as we discussed above. It was quite clear that the thresholds were much higher when pumped by the 355 nm laser compared to the 400 nm laser.

In summary, optically pumped GaN-based VCSELs with different cavity lengths were fabricated and characterized. The experimental results showed that with the cavity length reduced from 18 *λ* to 8.5 *λ*, the lasing threshold decreased, and meanwhile, the slope efficiency, spontaneous emission coupling factor and lasing linewidth increased. In addition, under the pumping of 355 nm laser, the lasing spectrum exhibited red shift and linewidth broadening with increased pumping energy due to the heating effect. Meanwhile, the lasing threshold was higher than that under 400 nm pumping. These results reveal that the performances of the GaN-based VCSELs are greatly influenced by cavity length and heating effect. Therefore, to control the cavity length and improve heat dissipation ability are particularly important for design and fabrication of GaN-based current injected VCSELs.

## Methods

### Device Fabrication

The VCSEL layer structure, grown on a 2-inch (0001) c-plane sapphire substrate by a metal-organic chemical vapor deposition (MOCVD) system, consists of a 30 nm GaN nucleation layer, a 2 μm undoped GaN layer, a 2 μm Si-doped GaN layer, MQWs active region, a 20 nm Mg-doped AlGaN electron blocking layer and a 380 nm Mg-doped GaN layer. The active region consists of ten periods of 2 nm InGaN quantum well and 18 nm GaN barrier. Fabrication of VCSELs was processed as follows. First, 12.5 pairs of Ta_2_O_5_/SiO_2_ layers were evaporated on the top of the grown VCSEL structure to form the first dielectric DBR. The reflectivity of the DBR R_1_ is about 99% at central wavelength. Next, the samples were bonded to a Si substrate. A pulsed excimer laser operating at a wavelength of 248 nm was used in the laser lift-off process to remove the sapphire substrate. After removal of residual Ga by HCl solution, ICP etching was carried out to remove the high dislocation region of undoped GaN layer and reduce the thickness of n-GaN layer. Then, three samples with different layer thicknesses were fabricated. The thicknesses of the residual epitaxial layer were controlled by the etching time of ICP. Afterwards, CMP technique was employed to polish the n-GaN surface using a colloidal silica material in alkaline environment. Finally, the top DBR consisting of 11 pairs of Ta_2_O_5_/SiO_2_ layers was deposited on the polished n-GaN surface. The reflectivity of the top DBR R_2_ is 97% around the central wavelength.

### Measurements

The lasing characteristics of the VCSELs were studied by either a 400 nm and or a 355 nm pumping laser. The 400 nm laser was obtained by taking the second harmonics of the 800 nm fundamental wave with ab-BaB_2_O_4_ (BBO) crystal. While the fundamental source was a regenerative amplifier (Spectra Physics, Spitfier), operating with a pulse width of 150 fs and a repetition rate of 1 kHz, seeded by a mode-locked Ti:sapphire laser. The 355 nm laser was with 25 ns pulse duration and 30 kHz repetition with the third-harmonics of a Q-switched YVO_4_ pulse laser. The pumping laser beams with focused spot size of 50 μm in diameter were incident normally on the VCSELs surface. The emission light was collected into a spectrometer using a grating of 1200 lines/mm with a spectral resolution of 0.15 nm.

## Author Contributions

B.Z. conceived the project. W.L, X.H., J.Z. and Z.C. fabricated the samples. S.C., W.L. and H.A. constructed the experimental apparatus. W.L., S.C. and L.Y. characterized the samples. W.L. and X.H. analyzed the data and wrote the manuscript under the supervision of B.Z. All authors participated in discussion and commented on the manuscript.
